# Outcome in juvenile idiopathic arthritis: a population-based study from Sweden

**DOI:** 10.1186/s13075-019-1994-8

**Published:** 2019-10-28

**Authors:** Elisabet Berthold, Bengt Månsson, Robin Kahn

**Affiliations:** 1 Department of Rheumatology, Clinical Sciences Lund, Lund University, 221 85 Lund, Sweden; 2 Department of Pediatrics, Clinical Sciences Lund, Lund University, Lund, Sweden; 3 Wallenberg Centre of Molecular Medicine, Lund University, Lund, Sweden

**Keywords:** JIA, Uveitis, Follow-up, Outcome, Incidence, Population-based

## Abstract

**Background:**

As the treatment arsenal for children with juvenile idiopathic arthritis (JIA) has expanded during the last decades, follow-up studies are needed on children diagnosed in the era of biological treatment to evaluate if this has improved the outcome. Our aim was to study the epidemiology and outcome of JIA in southern Sweden using a population-based cohort of children with a validated diagnosis of JIA collected over 9 years.

**Methods:**

Potential cases of JIA between 2002 and 2010 were collected after a database search, using the ICD codes M08-M09. The study area was Skåne, the southernmost county of Sweden (population 1.24 million; 17.6% aged < 16 years). The JIA diagnosis was validated and subcategorized through medical record review based on the criteria defined by the International League of Associations for Rheumatism (ILAR). Parameters on disease activity and pharmacologic treatment were recorded annually until the end of the study period (December 31, 2015).

**Results:**

In total, 251 cases of JIA were confirmed. The mean annual incidence rate for JIA was estimated to be 12.8/100,000 children < 16 years, with the highest age-specific annual incidence at the age of 2 years (36/100,000). Oligoarthritis was the largest subgroup (44.7%), and systemic JIA was the smallest subgroup (2.8%). Methotrexate was the most common disease-modifying anti-rheumatic drug prescribed (60.6%). Tumor necrosis factor alpha inhibitors were used as treatment for 23.9% of the children. Only 40.0% of the follow-up years, with a median follow-up time of 8 years, were free of arthritis or uveitis. Uveitis occurred in 10.8% of the children (8.0% chronic uveitis), and the need for joint corrective orthopedic surgery was 9.2%.

**Conclusions:**

The incidence of JIA in this well-defined, population-based cohort is slightly lower than in previously published studies from Scandinavia. The need for orthopedic surgery and the presence of uveitis are diminished compared to studies with patients diagnosed more than 20 years ago. Children with JIA however still experience disease activity more than 50% of the time. In conclusion, we still have long-term challenges in the care for children with JIA, in spite of state-of-the-art treatment.

## Background

Juvenile idiopathic arthritis (JIA) is the most common rheumatic disease in children, with an unpredictable clinical course and the impending risk of impaired joint function. JIA is an umbrella term encompassing a heterogeneous group of inflammatory arthritides of unknown etiology, all defined by the presence of at least one inflamed joint persisting ≥ 6 weeks, beginning before 16 years of age and that cannot be explained by other causes such as infection or trauma [1]. Historically, different names have been used for the disease, with juvenile rheumatic arthritis (JRA) with 6 weeks duration of arthritis being the American definition [2] and juvenile chronic arthritis (JCA) with arthritis enduring for at least 3 months being the European definition [3]. The present international consensus of JIA is based on the criteria defined by the International League of Associations for Rheumatism (ILAR) in 2001. According to these criteria, JIA is further divided into seven subcategories based on clinical features: enthesitis-related arthritis (ERA), oligoarticular JIA (persistent and extended), polyarticular rheumatoid factor-negative (RF−) JIA, polyarticular RF-positive (RF+) JIA, juvenile psoriatic arthritis (JPsA), systemic JIA (sJIA), and undifferentiated JIA (uJIA) [4].

The reports on incidence rates of JIA differ depending on the study design and geographic region. The worldwide incidence rate among Caucasians was in 2014 presented to be 8.3/100,000/year and the prevalence as 32.6/100,000/year [5]. These pooled rates were however based on studies using the three different disease classifications (ILAR, ACR, and EULAR). The incidence rates of juvenile arthritis differ between 15.0/100,000/year in the Nordic countries (ILAR) [6], 10.3/100,000 in Minnesota (USA) (ILAR and ACR) [7], 8.5/100,000 in Manitoba (Canada) (ILAR) [8], 6.9/100,000 in Catalonia (Spain) (ILAR) [9], and 3.1/100,000 in Alsace (France) (ILAR) [10].

The long-term outcome of JIA has improved in the last three decades [11]. There are different criteria developed to study and define disease outcome. In 2004, Wallace et al. defined a set of criteria for evaluation of clinical outcome in JIA. Inactive disease was defined as a state of no joints with active arthritis, no uveitis, no systemic symptoms, normal erythrocyte sedimentation rate (ESR) and/or C-reactive protein (CRP), and a physician’s global assessment of disease activity indicating no disease activity [12]. Using this definition, 47.5 ± 22.6% of the children achieved inactive disease after a median time of 6.5 ± 1.5 years, according to the data compiled in a review article on JIA outcome. Inactive disease and remission were achieved most often in the persistent oligoarticular subgroup, in contrast to the extended oligoarticular and RF+ subgroups, where the prognosis for achieving remission was least favorable [13]. In the most recently published outcome study, 45.6% of the children had active disease at 18 years of follow-up [14]. However, validated outcome criteria are difficult to use in a retrospective study due to the need for patient-reported measures.

Data on the rates of joint corrective surgery is sparse, but in a study on prevalent cases of JIA in Minnesota (USA) 1994–2013, 7% of the children with JIA had to undergo joint surgery during childhood with the same number needing it in adulthood [15]. In an older observational study on adult patients with JIA followed over a median time of 19 years, 28.5% had undergone joint surgery, with a majority needing joint replacement. Survival analysis showed that joint surgery was needed in more than 75% of the patients at 45 years disease duration [16].

Uveitis is the most common extra-articular manifestation of JIA, and the reported prevalence range from 11.6 to 30% [17]. In the prospective Nordic cohort study with JIA patients collected 1997–2000, uveitis occurred in 89 (20.5%) children, 80 chronic and 9 acute cases, during a median follow-up of 98 months. There were no uveitis cases among patients with systemic or RF+ JIA [18]. In Sweden, children with JIA have regular ophthalmologic controls ≥ 1 time per year until the age of 14, to discover chronic uveitis.

The aim of this study was to investigate the epidemiology and outcome of JIA and to characterize the demographics of the patients with JIA, using a well-defined population-based cohort of children with a validated diagnosis of JIA collected over 9 years.

## Methods

### Study area and population

The study area was Skåne, the southernmost county of Sweden, containing 33 municipalities with a total area of 11,027 km^2^ [19]. The population of the area was 1,243,329, 13.2% of the total Swedish population, by December 2010. Children (0–15 years) constituted 17.6% of the total population of Skåne with 219,330 individuals (48.6% females) in December 2010. Of these children, 24% were born outside of Sweden or in Sweden with 2 parents without Swedish heritage [20].

The healthcare system in Sweden is publicly financed. The care is subsidized for all children until at least 18 years of age and includes preventive standardized controls at a child health center and later at school healthcare, which diminishes the risk of missing a symptom of a disease such as JIA. There are no Swedish pediatric hospitals with exclusively private administration. The national diagnosis register is compulsory and independent of care facility. In the study area, there is 1 university hospital, 7 other hospital-associated pediatric outpatient facilities, and 8 private pediatric outpatient facilities. In Skåne, there are also 165 centers of primary care where children can receive healthcare. The center for pediatric rheumatology in Skåne is located at the University Hospital in Lund and receives patients from primary and secondary care, as well as tertiary referrals from the region and the neighboring healthcare regions.

### Case retrieval

The case retrieval process was a two-step procedure ensuring as close to the total coverage as possible. As the first step, a search for patients diagnosed with JIA between 2002 and 2010 was made in the clinical database at the local hospital register using the International Classification of Diseases (ICD) codes M08-M09 (ICD-10). The search was extended to children up to 18 years of age to secure any referred children with the diagnosis made in another healthcare facility. An additional search for total regional coverage was then made from the diagnosis register at the National Board for Health and Welfare (NBHW) using the same ICD codes recorded as primary as well as secondary diagnosis for outpatient and inpatient visits. Thus, to be included in the initial cohort, a patient only needed a single enrollment for inpatient care or one outpatient visit before 19 years of age, with a diagnosis code for JIA registered at any regional healthcare facility between 2002 and 2010.

Medical records for all the patients found were reviewed in order to establish a diagnosis of JIA according to the 2001 ILAR classification [4]. A patient was included in the study if diagnosed before the 16th birthday while living in Skåne between 1 January 2002 and 31 December 2010. At diagnostic uncertainty, we used a two-part consensus for diagnosis, and if that was not achieved, an additional experienced pediatric rheumatologist was consulted and diagnosis was set in consensus or by voting. The Regional Ethical Review Board for southern Sweden approved the study (2013/192 and 2015/62).

### Case ascertainment and classification

Due to the uncertainty in interpreting information about symptom debut, we stated the diagnosis date before the 16th birthday as the primary inclusion criterion. We defined diagnosis date as the date when a pediatric rheumatologist or a senior pediatrician with experience in the field of rheumatology coded the arthritis as JIA according to ICD-10. However, data from any years, starting from 2002, before the diagnosis date was included in the study if the patient, for example, had been controlled for “suspected JIA” and the diagnosis later was confirmed. The JIA diagnosis was further classified into subgroups according to the ILAR definition. We continuously reevaluated the diagnosis and classification during the entire study period, considering psoriasis, uveitis, and inflammatory bowel disease as a manifestation of the disease. Due to the retrospective data collection, hereditary information could only be used for classification when stated. The information of uveitis was ascertained based on the information stated in the pediatric review or when the medical review from an ophthalmologic hospital-based facility was available. The presence of RF at one occasion was used as an inclusion criterion for patients who otherwise met the criteria for polyarticular disease, as the local clinical guideline is to only test for RF once if it is present in a polyarticular disease. The presence of one positive RF was however not used as an exclusion criterion in patients with a pattern of oligoarticular disease.

### Collection and recording of data

For all patients, gender, age at diagnosis, disease onset (debut of symptoms), diagnosis date, presence of uveitis, immunological data on the presence of antinuclear antibodies (ANA), RF, anti-cyclic citrullinated peptide antibodies (aCCP), and HLAB27 were recorded. The following parameters were recorded annually: mean values of hemoglobin (Hb), white blood cell count (WBC), platelet count (PLT), ESR, and CRP; mean height and weight; orthopedic surgical procedures; and swollen and tender joint count. The joint count was recorded as the total number of individually affected joints in the 66/68 joint count index that year. Pharmacological treatment was also recorded annually and included even if only used for shorter periods. We did not register adverse events due to the uncertainty of the data since we only had information from the hospital medical records and not from the records in primary care. We did however actively search for cases of tuberculosis.

The patients were followed until the occurrence of death, migration from the study area, loss to follow-up, or end of the study on 31 of December 2015, whichever occurred first.

### Statistical analysis

We used conventional descriptive statistics such as absolute numbers, median, quartile range, and percentage to describe demographics and clinical outcome. The incidence rate was calculated using the number of incident cases as the numerator and the total pediatric population at risk during the study period as the denominator. For the calculation of age-specific incidence rates, the sum of children in each age during the study period was used as the denominator. Survival analysis on chronic uveitis, as well as the need for joint corrective surgery, was performed plotting a Kaplan-Meier survival curve for the whole group with the date of the first diagnosed uveitis/first surgical intervention as a failure point. We used years during the complete study period without arthritis and uveitis as our outcome measure for inactive disease and have presented it as the number of follow-up years without arthritis or uveitis, divided by the sum of follow-up years in the subgroup. We have excluded the first year of disease (year of diagnosis). Statistical analyses were performed using Statistical Package for Social Sciences software (SPSS 25.0 for Macintosh).

## Results

A total of 489 individuals with the ICD codes for JIA were found. Of these patients, 307 were confirmed to have JIA after a review of the charts. However, 56 patients were excluded, as their JIA diagnosis was later than the 16th birthday or their diagnosis was confirmed outside the study area. A total of 251 patients are thus remaining in the study cohort. Other reasons for exclusion were medical records not found (*n* = 5), other rheumatic diseases (*n* = 19), and non-rheumatic condition misdiagnosed as JIA (*n* = 158) (flow chart of the case collection procedure is enclosed as Additional file 1: Figure S1).

### Diagnosis distribution

Oligoarthritis is the largest subgroup (44.7%): persistent oligoarthritis 33.5% and extended oligoarthritis 11.2%, followed by uJIA (16.3%), RF− (13.9%), ERA (8.8%), RF+ (6.8%), JPsA (6.8%), and sJIA is the smallest subgroup (2.8%) (Table 1).

**Table 1:** Demographics of the JIA subgroups

	Total	ERA	Oligoarthritis	Extended	Persistent	RF−	RF+	JPsA	sJIA	uJIA
Number of patients (% of total)	251 (N/A)	22 (8.8%)	112 (44.6%)	28 (11.2%)	84 (33.5%)	35 (13.9%)	17 (6.8%)	17 (6.8%)	7 (2.8%)	41 (16.3%)
Female (%)	66.5	36.4	69.6	67.9	70.2	74.3	70.6	76.5	57.1	63.4
Age at debut (years (25–75 centiles))	7.3 (2.3–11.5)	10.7 (6.5–13.2)	4.0 (2.0–9.9)	6.4 (1.9–10.3)	3.7 (2.0–9.7)	5.6 (1.9–11.2)	12.7 (10.7–14.5)	9.7 (5.5–12.2)	8.3 (3.1–9.5)	6 (2.8–11.8)
Follow-up time (years (25–75 centiles))	8 (6–11)	7 (6–11)	8 (6–11)	10 (7–13)	8 (6–10)	8 (7–11)	9 (7–12)	8 (5.5–10)	4 (1–6)	9 (7–11)
Disease duration at diagnosis (months (25–75 centiles))	5 (2–14)	9 (3.5–23.5)	5 (2–14)	9 (3–27)	4 (2–10)	6 (2–13)	5 (2–23)	14 (5.5–47.5)	2 (0–4)	4 (1–8.5)
ANA (%)*	50.6 (100)	27.2 (100)	67.9 (100)	53.6 (100)	72.6 (100)	48.6 (100)	23.5 (100)	23.5 (100)	14.3 (100)	46.3 (100)
RF (%)*	10.8 (90.0)	0 (90.9)	4.5 (89.3)	14.3 (92.9)	1.2 (88.1)	0 (94.3)	100 (100)	5.9 (82.4)	0 (85.7)	9.8 (87.8)
aCCP (%)*	6.8 (66.1)	4.5 (77.3)	1.8 (63.7)	7.1 (67.9)	0 (61.9)	2.9 (80.0)	70.6 (94.1)	0 (64.7)	0 (28.6)	2.4 (51.2)
HLAB27 (%)*	14.7 (45.0)	81.8 (90.9)	4.5 (34.8)	3.6 (42.9)	4.8 (32.1)	8.6 (54.3)	0 (35.3)	5.9 (41.2)	0 (14.3)	24.4 (51.2)

*Data coverage presented in the parenthesis

### Incidence rate

The mean annual incidence rate for JIA was estimated to be 12.8 (95% confidence interval 11.3–14.5) per 100,000 children < 16 years. In females, the mean annual incidence rate was 17.5 (15.0–20.4) per 100,000, and in males, the corresponding number was 8.3 (6.7–10.3) per 100,000.

**Fig. 1:**
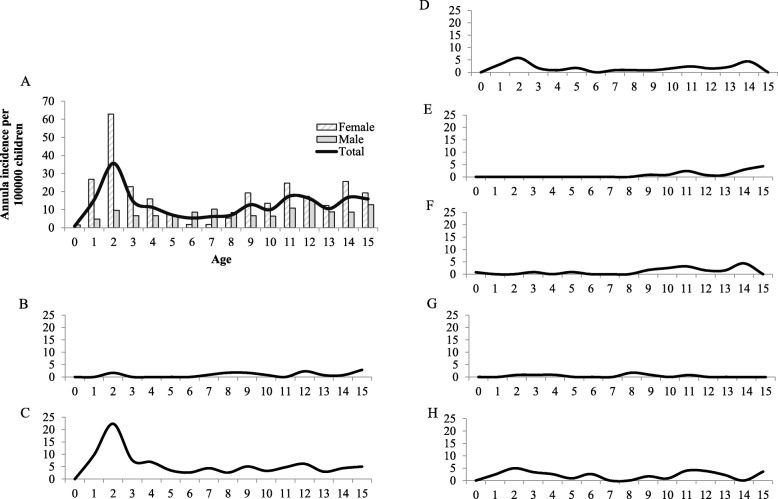
Mean annual incidence rate. **a** The bar chart shows the age-specific mean annual incidence rate divided by gender, presented per 100,000 children. The line shows the age-specific incidence rate of the total cohort. **b**–**h** The line charts visualize the age-specific annual incidence rates per 100,000 children in the diagnostic subgroups. **b** Enthesitis-related arthritis (ERA). **c** Oligoarthritis. **d** RF-negative polyarthritis (RF−). **e** RF-positive polyarthritis (RF+). **f** Juvenile psoriatic arthritis (JPsA). **g** Systemic juvenile idiopathic arthritis (sJIA). **h** Undifferentiated juvenile idiopathic arthritis (uJIA)

When studying the age-specific annual incidence rates, the peak is at 2 years, 36/100,000. This peak is consistent in the female group, but the incidence peak among males is not until 12 years (Fig. 1a).

We further investigated the incidence rate for the different subgroups. The oligoarticular subgroup has the highest incident rate in younger children, whereas the incidence rates for ERA and RF+ peak in the older age groups. The subgroups of uJIA and RF− have a more evenly distributed age-related incidence rate (Fig. 1b).

### Demographics

In the total cohort, 2/3 of the children are female and it is only in the ERA group where males predominate. The median age at diagnosis in the cohort is 7.3 years; the highest median age of 12.7 is in the RF+ group and 10.7 years in the ERA group. The children are followed up for a median of 8 years. The median time between symptom debut and specialist diagnosis is 5 months; in the JPsA group, this duration is as long as 14 months.

Half of the children in the population are ANA-positive, and among them, 59.8% have oligoarthritis. RF is present in 10.8% of the population on at least one occasion; 63.0% of these patients have a polyarticular JIA; 14.7% are carriers of HLAB27; 48.6% of them have ERA (Table 1).

### Pharmacological treatment

The distribution of pharmacological treatment is presented in Table 2. The category “no treatment” refers to a year with no pharmacological treatment, i.e., a patient has discontinued all treatment during one calendar year. Only 43.3% of the patients met this criterion at some point during the follow-up. Almost all patients (98%) are at some point during their disease course prescribed non-steroid anti-inflammatory drugs (NSAID). Local, intra-articular steroid injections are also often used (78.9%) and are, except in the systemic group, relatively equally often used in the different subgroups. Methotrexate is the most common disease-modifying anti-rheumatic drug (DMARD) prescribed (60.6%). It is used by all children with RF+ disease and is a more common treatment option in all the polyarticular groups. Tumor necrosis factor alpha (TNFα) inhibitor is used as a treatment in 23.9% of the children, predominantly with polyarticular disease and in 83.3% in combination with methotrexate. Other DMARDs, conventional as well as biologic, have also been used as a treatment. However, all patients with other biological DMARDs have also tried at least one TNFα inhibitor. No cases of active tuberculosis were found after the initiation of biological DMARDs (a more detailed table with treatment options is enclosed as Additional file 2: Table S1).

**Table 2:** Pharmacological treatment

	Total	ERA	Oligoarticular		RF−	RF+	JPsA	sJIA	uJIA
			Extended	Persistent					
No treatment	109 (43.4)	6 (27.3)	13 (46.4)	49 (58.3)	11 (31.4)	4 (23.5)	7 (41.2)	5 (71.4)	14 (34.1)
NSAID	246 (98.0)	22 (100)	27 (96.4)	83 (98.8)	35 (100)	16 (94.1)	17 (100)	7 (100)	39 (95.1)
Oral glucocorticoids	107 (42.6)	11 (50)	14 (50)	12 (14.3)	21 (60)	14 (82.4)	7 (41.2)	5 (71.4)	23 (56.1)
Intra-articular steroids*	198 (78.9)	16 (72.7)	25 (89.3)	69 (82.1)	25 (71.4)	15 (88.2)	11 (64.7)	3 (42.9)	34 (82.9)
cDMARDs	163 (64.9)	17 (77.3)	25 (89.3)	27 (32.1)	32 (91.4)	17 (100)	12 (70.6)	2 (28.6)	32 (78.0)
bDMARD	60 (23.9)	7 (31.8)	11 (39.2)	3 (3.6)	13 (37.1)	12 (70.6)	2 (11.8)	1 (14.3)	11 (26.8)
TNFα inhibitor + methotrexate	52 (20.7)	4 (18.2)	11 (39.2)	1 (1.2)	13 (37.1)	12 (70.6)	2 (11.8)	1 (14.3)	8 (19.5)

Numbers are presented as *n* with the percentage of the children in the subgroup in the parentheses. The numbers represent a treatment year in one patient

*Intra-articular glucocorticoid injections were considered as a treatment entity of its own. Thus, we have not taken into account the numbers of injections per year in a single patient

### Outcome

**Fig. 2:**
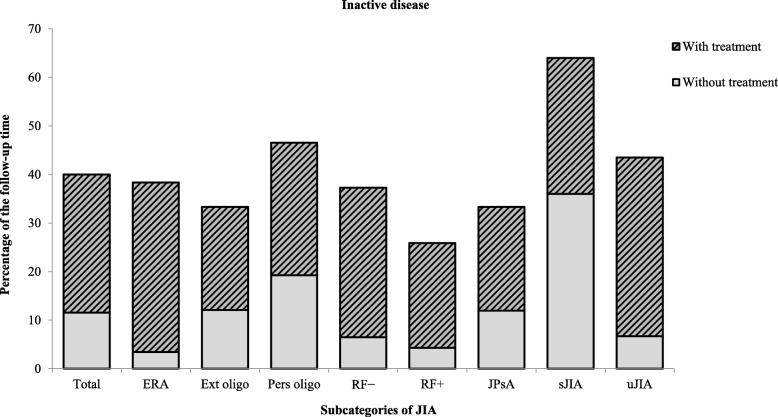
Inactive disease. Inactive disease was defined as a year without arthritis or uveitis. The bars represent the years with inactive disease presented as the percentage of the total follow-up time (years) in every subgroup. The light gray areas represent the years with inactive disease without any pharmacological treatment, and the striped areas represent the years with inactive disease on medication

In the entire cohort of children with JIA, consisting of all subgroups in the total follow-up period, 40.0% of the years were with inactive disease (defined as no arthritis or uveitis), 54.8% were active due to arthritis with or without uveitis, and 5.2% were active because of uveitis only. The median follow-up time was 8.0 years. In the subgroups, the percentages of inactive disease presents as follows: ERA 38.4%, oligoarthritis 42.5% (with extended oligoarthritis 33.3% and persistent oligoarthritis 46.5%), RF− 37.3%, RF+ 25.9%, JPsA 33.3%, sJIA 64.0%, and uJIA 43.5%. 28.8% of the inactive years were without treatment (percentage presented as gray bars) (Fig. 2). One patient that was lost to follow-up was later found out to have died.

**Fig. 3:**
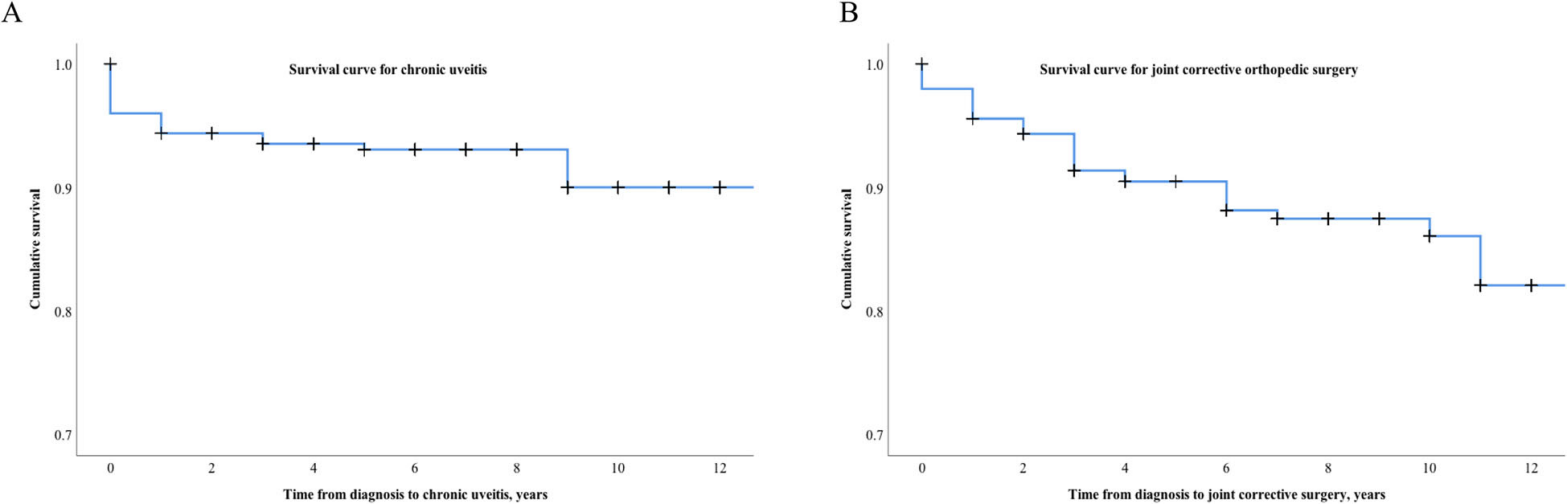
Chronic uveitis and joint corrective orthopedic surgery survival analysis. Survival curve according to Kaplan-Meier analysis. **a** First time chronic uveitis is present in 50% of the children the first year of disease but may occur throughout the entire follow-up time. Children with acute uveitis are not included in the analysis. **b** Joint corrective orthopedic surgery occurs throughout the entire follow-up period in JIA according to Kaplan-Meier analysis. At the end of the study period, 9.2% had been treated with joint corrective surgery

Uveitis was seen in 27 (10.8%) of the children, 8.0% had chronic uveitis, and 4.0% had acute uveitis (3 individuals have had both manifestations). Fourteen of the children have had uveitis in their debut year (10 chronic). The median debut age of chronic uveitis is 5.5 years (range 0–16 years). There are no cases of uveitis in the RF+, JPsA, or sJIA groups (Table 3). The risk of chronic uveitis is 10.0% at 12 years of follow-up using Kaplan-Meier survival analysis (Fig. 3a).

**Table 3:** Outcome in the subgroups

	Total	ERA	Oligoarthritis	Extended	Persistent	RF−	RF+	JPsA	sJIA	uJIA
Acute uveitis (%)	4	31.8	0	0	0	0	0	0	0	7.3
Chronic uveitis (%)	8	9.1	8.9	10.7	8.3	17.1	0	0	0	4.9
Orthopedic joint corrective surgery* (%)	9.2	4.5	8.9	17.8	6	5.7	23.5	17.6	0	7.3

The numbers are percentage of the individuals in each subgroup

*Eight individuals have undergone multiple joint corrective surgeries: six with two surgeries, one with three surgeries, and one with four surgeries (both of the latter with RF+ disease)

In total, 23 (9.2%) individuals have been treated with joint corrective orthopedic surgery, 8 of them with multiple procedures (3 with RF+ JIA, 2 with oligoarticular disease, 1 with RF− JIA, 1 with JPsA, and 1 with uJIA) (Table 3). However, only 11 individuals (4.4%) have undergone large orthopedic surgery (arthroplasty, osteotomies, or arthrodesis). The procedures were 17 synovectomies (5 with diagnostic purpose), 7 arthrodeses, 6 osteotomies, 4 medial knee epiphysiodeses, 1 arthrolysis, 1 arthroplasty (hip prosthesis), 1 volar tenosynovectomy, and 1 finger tendon transposition. The need for orthopedic surgery is the highest (23.5%) in the group with RF+ JIA. The risk for joint corrective surgery is 17.9% at 12 years of follow-up using Kaplan-Meier survival analysis (Fig. 3b). The risk for a serious orthopedic procedure is 9.4% at 12 years of follow-up.

## Discussion

We have investigated the long-term outcome of JIA using this well-defined, population-based cohort of 251 children from southern Sweden with a validated diagnosis of JIA, all diagnosed in the era of biologic treatment. The mean annual incidence rate for JIA was 12.8 per 100,000 children. A considerable part of the children (10.8%) still develop uveitis, and the majority of the follow-up years (60.0%) are spent in a state of active disease. However, only 4.4% of the children require serious orthopedic corrective surgery such as arthrodesis, osteotomy, or arthroplasty.

We have found an annual incidence rate of 12.8 (95% confidence interval 11.3–14.5) per 100,000 children. This is a lower number than in previously published studies from Sweden and the Nordic countries (15/100,000) [6]. However, our case collection process, with the collection of ICD codes covering arthritic and psoriatic diagnoses in childhood from the local diagnosis register as well as from the NBHW, diminishes the possibility of selection and referral bias.

Our incidence rate is also close to the incidence rate of 14/100,000, presented in a prospective population-based study carried out in the southeastern part of Norway, with almost an equal amount of children at risk as in our study area of southern Sweden. However, the purpose of the Norwegian study was to study the incidence of all arthritides in childhood [21]. The incidence rate of JIA published from Olmsted County, MN, 10.3/100,000 [7], is also close to our number. However, as we move down to southern Europe, the incidence rates of 6.9/100,000 in Catalonia (Spain) [9] and 3.1/100,000 in Alsace (France) [10] are distinctly lower than in our cohort. When looking at our results together with these selected previously published incidence rates, we bring validation to the suggestion that there seems to exist a north-south gradient in the incidence of JIA in Europe [22]. Genetic and environmental factors, as well as infectious agents, are risk factors for developing JIA, and a geographical gradient explains the combined impact of these factors.

With the retrospective approach of our study, we have the challenge of working with information already stated in the medical review, making it sometimes difficult to strictly apply the exclusion criteria stated in the ILAR definitions to the characterization of a patient. As presented in the “Methods” section, we used the presence of RF at one occasion as an inclusion criterion for patients who otherwise met the criteria for polyarticular disease, but not as an exclusion criterion in patients with a clinical manifestation of oligoarticular disease. This might give an overestimation of the RF+ group and a lesser portion of patients in the uJIA group, and there is also a risk of underestimation of patients in the uJIA group due to missing information about heredity. However, the diagnosis distribution in our cohort is similar to that in the Canadian ReAACh-out cohort, a large prospective JIA cohort for the purpose of studying the outcome in JIA patients. The ReAACh-out cohort comprises approximately 40% oligoarticular disease and 25% polyarticular disease but a larger amount of ERA (14.2% vs. 8.8%) and a smaller amount of uJIA (10% vs. 16.3%) than in our cohort [23]. The diagnostic subgroups in our cohort are also similar to the distribution in the Nordic cohort [6] except that they have classified 22% of the patients (vs. 16.3% in our study) as uJIA, suggesting that our possible lack of hereditary information is of some importance. The demographic information in our cohort is consistent with that in the cohorts mentioned above. In the ReAACh-out cohort, the median time from disease start to diagnosis was 4.3 months [23], as compared to 5.0 months in our study and 6.6 months in the Olmsted county cohort [7]. A new set of classification criteria for JIA has been proposed [24]. How this will influence the outcome in the subgroups is uncertain. However, it could be of interest to see what impact these new criteria will have on the outcome in our current subgroups as well as to validate the criteria in this cohort.

The characterization of the medical treatment in our cohort shows that almost all children (98%) are prescribed NSAID. However, not only continuous treatment is registered but also NSAID prescribed to be taken in case of disease activity. Intra-articular corticosteroid injections were also an often-used treatment option in this cohort (78.9%), consistent with the current treatment recommendations. Due to the risk of growth retardation and other adverse effects with systemic glucocorticoids, intra-articular injections are favorable. Despite this fact, 42.6% of the total cohort is prescribed systemic glucocorticoids, but on 60.7% of these occasions, it was prescribed for a shorter duration than 2 months. A positive discovery was that 43.4% of the patients experience years of medication, six out of ten of the children with persistent oligoarthritis. Our results on medical treatment are in line with the results from another published prospective cohort from the Nordic countries (except for Iceland) with children included during 1997–2000 [25]. As many as 96.1% of the children in this cohort were treated with NSAID and 74.1% received intra-articular corticosteroid injections. The treatment with methotrexate and TNF inhibitors was somewhat more unusual than in our cohort, 48.4% vs. 60.6% for methotrexate and 17.5% vs. 23.9% for TNF inhibitors, which we interpret as an effect of the 10-year difference of the inclusion periods.

Even though these patients all have been diagnosed with JIA in the treatment era of biological DMARDs, the presence of uveitis is 10%, the need for serious orthopedic joint corrective surgery is 4.4%, and 60% of the follow-up years are with active disease. The presence of uveitis in our cohort is however half of the prevalence in the Nordic cohort study with a corresponding number of median follow-up years [18], which also in this respect interprets as an effect of the 10-year difference of inclusion period and the more common use of DMARDs in our cohort. There were no cases of uveitis in the RF+, systemic, or psoriatic subgroups in our cohort as well, which raises the question of the need for regular ophthalmologic controls in these groups of children. Most cases of chronic uveitis occur in the first 3 years of disease, but our data show that it still can develop after 9 years, at a time when Swedish regular ophthalmologic controls are sparse to once or twice per year, or even finished for most patients. Thus, we conclude that there is no absolute time or age limit to the end of risk for developing JIA-associated chronic uveitis.

It also seems that children with JIA today are in need of joint corrective surgery to a lesser extent than 20 years ago [16], as a suggestive proof of more frequent use of methotrexate and the biologic DMARDs being effective in diminishing long-term effects of the disease. However, the children in our cohort have spent the majority of their time with inflammatory activity in the joints or eyes. This might partly be an effect of our decision to present the swollen and tender joint count as the total number of affected joints in the 66/68 joint count index that year, i.e., 1 minor arthritis at 1 check-up visit is considered as disease activity that year, but it is also close to the finding that only 47.5% of JIA patients achieve inactive disease at a median of 6.5 years [13]. Thus, a surprisingly large amount of children with JIA still do not achieve inactive disease with the arsenal of treatment options available today, but on the other hand, the functional impact is less evident than 20 years ago. One way to simplify the outcome presentation would have been to use JADAS measures to describe disease activity and disease remission, but this was unfortunately not possible because of the lack of both the parameters of physician global assessment of disease activity and parents/patient global assessment of well-being due to the retrospective study design.

There are limitations to our study. The most important limitation is the retrospective nature, with possible consequences discussed above*.* On the other hand, the strengths of our study include the population-based approach with minimal or no selection bias and inclusion of patients from all regional healthcare providers. The JIA diagnosis is validated for every patient, and the same training physician has made the validation, also diminishing inclusion bias. It is interesting to point out that as many as 32% of the cases were excluded as they were misdiagnosed as JIA. Of course, a part of the cases were arthritides diagnosed with an ICD code for JIA and labeled “suspected JIA” and later in the medical history reclassified as for example post-infectious arthritis, but a considerable part of these excluded cases turned out to be other conditions incorrectly coded and registered as JIA. In the study of incidence and prevalence of JIA in California (USA), Harrold et al. reviewed a random sample of medical records registered as JIA in order to develop the best case-finding algorithm for the study purpose. Out of the 97 selected records, 69% were determined to have JIA [26]. Thus, when using population-based cohorts of a disease without validation of the individual cases for the purpose of studying causality, the risk of diagnosis misclassification has to be taken into account when making conclusions on the result. The excluded number due to misclassification was higher than expected in our study.

## Conclusions

The annual incidence rate of JIA in southern Sweden is 12.8/100,000 children < 16 years. More importantly, we show that even in children diagnosed in the era of biologics and treat-to-target strategies, only 40% of the follow-up years are free of arthritis or uveitis. However, fewer patients develop uveitis (10.8%) and are in need of serious orthopedic surgery (4.4%) than previously published data on children diagnosed with JIA 20 years ago.

This study, to our knowledge the only retrospectively validated population-based cohort published, highlights the need for further clinical studies aiming to improve the care for children with JIA.

## Supplementary information

**Additional file 1:** **Figure S1.** Case collection procedure. (DOCX 63 kb)

**Additional file 2.** Supplementary table of treatment options. (PDF 56 kb)

## Data Availability

The datasets generated and analyzed during the current study are not publicly available due to them containing information that could compromise research participant privacy. The data are available and anonymized from the corresponding author (EB) on reasonable request.

## Abbreviations

JIA: Juvenile idiopathic arthritis
ICD: International Classification of Diseases
ILAR: International League of Associations for Rheumatism
JRA: Juvenile rheumatic arthritis
JCA: Juvenile chronic arthritis
ERA: Enthesitis-related arthritis
RF: Rheumatoid factor
RF−: RF-negative polyarticular
RF+: RF-positive polyarticular
JPsA: Juvenile psoriatic arthritis
sJIA: Systemic arthritis
uJIA: Undifferentiated arthritis
ESR: Erythrocyte sedimentation rate
CRP: C-reactive protein
NBHW: National Board for Health and Welfare
ANA: Antinuclear antibodies
aCCP: Anti-cyclic citrullinated peptide antibodies
Hb: Hemoglobin
WBC: White blood cell count
PLT: Platelet count
SPSS: Statistical Package for Social Sciences
NSAID: Non-steroid anti-inflammatory drug
DMARD: Disease-modifying anti-rheumatic drug
TNFα: Tumor necrosis factor alpha
