# Supplementary material for: Outcome in juvenile idiopathic arthritis: a population-based study from Sweden
Source: Arthritis Res Ther. 2019 Oct 28;21:218. doi: 10.1186/s13075-019-1994-8 (PMC6816211; doi:10.1186/s13075-019-1994-8)
Supplement: Supplementary file 1 — Additional file 1: Figure S1. Case collection procedure. (DOCX 63 kb) [file 13075_2019_1994_MOESM1_ESM.docx]

**Additional file 1: Figure S1: Case collection procedure.**

Patients included in the cohort were to be diagnosed with JIA 2002-2010 in Skåne before the age of 16. The diagnoses were collected from the local hospital register and the National Board for Health and Welfare (NBHW) using the ICD-codes M08-09. The cases were excluded due to the reasons stated above.
