# Supplementary material for: Outcome in juvenile idiopathic arthritis: a population-based study from Sweden
Source: Arthritis Res Ther. 2019 Oct 28;21:218. doi: 10.1186/s13075-019-1994-8 (PMC6816211; doi:10.1186/s13075-019-1994-8)
Supplement: Supplementary file 2 — Additional file 2. Supplementary table of treatment options. (PDF 56 kb) [file 13075_2019_1994_MOESM2_ESM.pdf]

Supplementary table 1

				Oligoarticular						
		**Total (n 251)**	**ERA (n 22)**	**Extended (n 28)**	**Persistent (n 84)**	**RF− (n 35)**	**RF+ (n 17)**	**JPsA (n 17)**	**sJIA (n 7)**	**uJIA (n 41)**
	**No treatment**	109 (43.4)	6 (27.3)	13 (46.4)	49 (58.3)	11 (31.4)	4 (23.5)	7 (41.2)	5 (71.4)	14 (34.1)
	**NSAID**	246 (98.0)	22 (100)	27 (96.4)	83 (98.8)	35 (100)	16 (94.1)	17 (100)	7 (100)	39 (95.1)
**Glucocorticoids**	**Oral**	107 (42.6)	11 (50)	14 (50)	12 (14.3)	21 (60)	14 (82.4)	7 (41.2)	5 (71.4)	23 (56.1)
	**Oral < 2 months**	65 (25.9)	8 (36.4)	12 (42.9)	11 (13.1)	14 (40)	8 (47.1)	2 (11.8)		10 (24.4)
	**Intra-articular steroids**	198 (78.9)	16 (72.7)	25 (89.3)	69 (82.1)	25 (71.4)	15 (88.2)	11 (64.7)	3 (42.9)	34 (82.9)
**sDMARD**	**Methotrexate**	152 (60.6)	15 (68.1)	23 (82.1)	27 (32.1)	32 (91.4)	17 (100)	11 (64.7)	2 (28.6)	25 (61.0)
	**Salazopyrine**	25 (10.0)	4 (18.2)	3 (10.7)	1 (1.2)	3 (8.6)	1 (5.9)	1 (5.9)		12 (29.3)
	**Azatioprine**	8 (3.2)	2 (9.1)	1 (3.6)		1 (2.9)	1 (5.9)			3 (7.3)
	**Antimalarial drugs**	17 (6.8)	1 (4.5)	6 (21.4)		2 (5.7)	2 (11.8)	1 (5.9)		5 (12.2)
**bDMARD**	**TNFα-inhibitor**	60 (23.9)	7 (31.8)	11 (39.2)	3 (3.6)	13 (37.1)	12 (70.5)	2 (11.8)	1 (14.3)	11 (26.8)
	**TNFα-inhibitor + methotrexate**	52 (20.7)	4 (18.2)	11 (39.2)	1 (1.2)	13 (37.1)	12 (70.6)	2 (11.8)	1 (14.3)	8 (19.5)
	**Adalimumab**	27 (10.8)	4 (18.2)	4 (14.3)	2 (2.4)	7 (20.0)	4 (23.5)	2 (11.8)		4 (9.8)
	**Etanercept**	44 (17.5)	3 (4.5)	11 (39.2)	2 (2.4)	7 (20.0)	9 (52.9)	2 (11.8)	1 (14.3)	9 (22.0)
	**Infliximab**	14 (5.6)	3 (4.5)			2 (5.7)	4 (23.5)	1 (5.9)	1 (14.3)	3 (7.3)
	**Anakinra**	2 (0.8)	1 (4.5)						1 (14.3)	
	**Abatacept**	3 (1.2)	2 (9.1)	1 (3.6)						
	**Tocilizumab**	7 (2.8)	2 (9.1)	2 (7.1)		1 (2.9)			1 (14.3)	1 (2.4)
